# Influence of environmental settings, including vegetation, on speciation of the redox-sensitive elements in the sediments of monomictic Lake Kinneret

**DOI:** 10.1007/s10201-024-00756-7

**Published:** 2024-07-22

**Authors:** Alexey Kamyshny, Rotem Klein, Werner Eckert, Khoren Avetisyan

**Affiliations:** 1 Department of Geological and Environmental Sciences, Faculty of Natural Sciences, Ben-Gurion University of the Negev, P.O. Box 653, 84105 Beer Sheva, Israel; 2 Yigal Allon Kinneret Limnological Laboratory, Israel Oceanographic & Limnological Research Ltd, P.O. Box 447, 14950 Migdal, Israel; 3 Department of Physical and Environmental Sciences, University of Toronto Scarborough, 1265 Military Trail, Toronto, ON M1C 1A4 Canada

**Keywords:** Lake Kinneret, Roots, Bioturbation, Sulfur, Iron, Manganese

## Abstract

The redox conditions in the littoral limnic sediments may be affected by the penetration of plant roots which provide channels for oxygen transport into the sediment while decomposition of the dead roots results in consumption of oxygen. The goal of this work was to study the impact of environmental parameters including penetration of roots of *Cyperus articulatus* L. into the sediments on cycling of the redox-sensitive elements in Lake Kinneret. We measured roots content, porosity, and chemical parameters including pH, sulfur, iron and manganese speciation in the sediments from the shore, littoral and sublittoral zones with and without vegetation. Our results show that at ≥ 12 m water depth, the upper 10 cm of the sediments are affected by the active sulfur cycling with concentrations of hydrogen sulfide > 70 μM near the sediment–water interface. Speciation of sulfur, iron, and manganese in the upper 10 cm of littoral sediments, which are covered by < 20 cm of water, are affected by their permeability and, to a lesser extent, by roots penetration. In the case when sediments are not covered by water, oxygen penetration to the sediments by desiccation is an additional important control of the redox zonation in the surface sediments. In the shore sediments, despite relatively high concentrations of sulfate in the pore-waters, sulfur cycling may be described as “cryptic” as expressed by very low concentrations of hydrogen sulfide in the pore-waters. This is most likely a result of its fast reoxidation by the abundant highly reactive Fe(III) and Mn(IV) phases.

**Supplementary Information:**

The online version contains supplementary material available at 10.1007/s10201-024-00756-7.

## Introduction

Vertical redox gradient in the sediments is controlled by the availability of electron donors and acceptors and consequently are strongly affected by bioturbation and bioirrigation (Orcutt et al. 2011). Anaerobic respiration processes utilize electron acceptors, which are energetically less favorable than oxygen for oxidation of organic matter and inorganic electron donors. These electron acceptors include nitrate, Mn(IV), Fe(III), sulfate and carbon dioxide (Jørgensen 2021). Nitrate is usually consumed immediately below the oxic zone due to its low concentrations, and Mn(IV) reduction starts just below the oxic zone in the sediments followed by Fe(III) and sulfate reduction zones (Froelich et al. 1979). The latter three processes often overlap spatially (Canfield et al. 1993; Hansel et al. 2015; Thamdrup 2000). Due to its high abundance, Fe(III) may be responsible for a large fraction of anaerobic oxidation of organic matter in the limnic sediments. For example, Fe(III) reduction is responsible for the 44% of total organic matter mineralization in deep Lake Michigan (Thomsen et al. 2004). In freshwater systems which contain less than 1 mM of sulfate, it becomes depleted and accounts for a smaller fraction of organic matter mineralization than in the marine sediments. The accumulation of hydrogen sulfide in the pore-waters is controlled by concentration of reactive iron, which precipitate hydrogen sulfide in the form of amorphous iron sulfide minerals, and pyrite as the final product (Rickard and Luther 2007). The methane-rich zone is formed at depths where sulfate is exhausted and methanogenesis leads to accumulation of methane in deep sub-surface sediments (Jørgensen and Kasten 2006). A similar redox cascade may develop in the water columns of stratified lakes, in which anoxic hypolimnion may contain either dissolved Fe(II) (Boyko et al. 2021; Crowe et al. 2008; Schiff et al. 2017) and Mn(II) (Findlay et al. 2019) or hydrogen sulfide (Avetisyan et al. 2021; Canfield et al. 2010; Kamyshny et al. 2011; Knossow et al. 2015; Zerkle et al. 2010) depending on the fluxes of iron, manganese and sulfur to the lake.

Bioturbation is a result of activity of different species including burrowing animals and rooting plants (Meysmann et al. 2005, 2006, 2010; Volkenborn and Reise 2006; Volkenborn et al. 2007). Submerged plants and plant roots are adapted to survive at both oxic and anoxic conditions due to the diurnal as well as seasonal variability in the redox conditions (Barko et al. 1991). Penetration of the roots into the sediments has two opposite effects on the redox conditions: transport of oxygen to the anoxic sediment layers through the roots and formation of more reduced sedimentary environment due to release of organic matter from the roots and decomposition of the dead roots. Some plants, for example *Spartina alterniflora* (Loisel.) P. M. Peterson and Saarela and *Spartina anglica* C. E. Hubb are known to actively pump oxygen through their root and oxygenate surrounding sediment in order to survive in sulfidic environment (Dias et al. 2016; Koop-Jacobsen and Wenzhofer 2015; Koop-Jacobcen et al. 2017; Lee et al. 1999). Although no experiments on transport of oxygen into the sediments were performed with *Cyperus articulatus* L., which populates the shores of Lake Kinneret, other *Cyperus* species are known to release oxygen from their roots into the anoxic sediments (Sorrell et al. 1993), artificial wastewater (Yao et al. 2011), constructed wetland (Cheng et al. 2014), and hydroponic culture (Manzur et al. 2015). Oxygen transport to the deep sediment layers leads to acceleration of organic matter degradation and oxidizes reduced solutes and minerals in the pore-water and sediment. In low-sulfate lakes, where methanogenesis is a quantitatively important process in the sediments, plant species are adapted to cope with methane presence. For example, rice plants transport methane from the rhizosphere to the atmosphere (Nouchi et al. 1990). On the other hand, both living and dead roots may serve as a source of organic matter in the sediments. Living roots and rhizomes supply organic matter to the sediment through leakage of dissolved organic compounds into the sediments (Hines et al. 1989; Mendelssohn et al. 1981). This process provides a labile substrate that can be used by sulfate reducing bacteria (Ferreira et al. 2007; Gribsholt et al. 2003). For example, it was found that certain plants release ethanol to the rhizosphere under hypoxic conditions (Smits et al. 1990). The dead roots and rhizomes are a significant but relatively recalcitrant source of organic carbon for microbial mineralization processes (Schubauer and Hopkinson 1984). The decomposition of plants releases mainly polysaccharides and lignin as well as aliphatic biopolymers and tannins (Kögel-Knabner 2002). Formation of reducing environments in the sediments due to the decomposition of plant material is well documented (Böttcher et al. 1998; Rusch et al. 1998). Oxygen uptake during decomposition of *Cyperus* species was quantified by Bianchini et al. (2008, 2011).

This study was designed to elucidate the effects of water depth, season, sediment properties and the presence of vegetation on the speciation and cycling of the redox-sensitive elements in the sediments of monomictic Lake Kinneret. Toward this goal, we have measured concentrations of sulfur, iron and manganese species in the pore-waters and solid phase of the sediments during seven sampling campaigns at the deepest part of the lake, at intermediate depth and in the littoral sediments covered and not covered by water and vegetation.

## Study site

A warm monomictic Lake Kinneret (The Sea of Galilee, northern Israel) is located in the northern part of the Syrian-African Rift Valley (32°50′N, 35°35′E), 210 m below the sea level. Its maximum and mean water depths are 43 m and 24 m, respectively, and surface area 168 km^2^ (Serruya 1978). The major contributory streams of the lake include freshwater from Jordan River and saline springs (Serruya 1978). The lake lies in the Mediterranean climate zone, with hot, dry summers and cool, wet winters with rainfall limited to November—March (Berman et al. 2014). Lake Kinneret is thermally stratified from the spring (March–April) to winter (December-January) (Rimmer et al. 2011). During the stratified period, the depth of the chemocline raises from 30–35 m in May to 12–18 m in summer and begins to drop in autumn until full mixis of the lake in winter (Avetisyan et al. 2019; Knossow et al. 2015). The hypolimnetic temperature is 14–16°C (Avetisyan et al. 2019; Knossow et al. 2015). The conductivity of water column varies between 1000 and 1300 μS cm^−1^ and is higher in the hypolimnion than in the epilimnion (Knossow et al. 2015). The salinity of the water column is 241 ± 25 mg L^−1^ Cl^−^ (Rimmer and Nishri 2014). In the epilimnion, the concentrations of soluble reactive phosphorus are usually < 0.2 μM at all seasons. With the onset of thermal stratification, the hypolimnetic concentrations of soluble reactive phosphorus and ammonium increases with time after stratification and is up to 2.5 μM and 110 μM, respectively, until the mixis (Berman et al. 2014). Ammonium accumulates in the hypolimnion during the stratified period in summer–fall reaching concentrations of 110 μM. Nitrate concentrations increase during the destratification of the water column due to nitrification of the hypolimnetic ammonium and during winter due to riverine inflows in winter and spring and nitrification of ammonium previously accumulated in the hypolimnion (Berman et al. 2014). A steep rise of chlorophyll concentrations in the water column occurs in winter and early spring with the highest content in April. Between the late spring and winter, the chlorophyll content is lower and is usually at the levels of 120 ± 30 mg Chl m^−2^ (Berman et al. 2014).

As a result of raising temperature and nutrients concentrations due to the mixing of the water column and influx of water from winter floods in spring, cyanobacterial and algal blooms occur in late winter and spring (Berman et al. 1992; Nino et al. 2020). As a result, fluxes of organic carbon to the hypolimnion increases during this season (Hadas and Pinkas 1995). Following stratification, the hypolimnion undergoes a sequence of redox changes as a result of the succession of microbially mediated processes, including oxygen consumption, denitrification and sulfate reduction (Cavari and Phelps 1977; Hadas and Pinkas 1995). Sulfate concentration in epilimnion of Lake Kinneret is 0.5–0.6 mM and decreases to < 0.3 mM in the hypolimnion due to microbial sulfate reduction (Knossow et al. 2015). The occurrence of hydrogen sulfide during early summer typically leads to metalimnetic blooms of phototrophic sulfur bacteria (Bergstein et al. 1979; Eckert et al. 1990). Active sulfur cycling in the chemocline of the lake was documented by Avetisyan et al. (2019). An important factor which affects sulfur biogeochemical transformations at the chemocline is internal waves or seiches. Impact of seiches on the biogeochemical sulfur cycling in the chemocline of the lake was shown in numerous works (e.g., Avetisyan et al. 2019; Boegman et al. 2003; Ostrovsky 1996; Rimmer et al. 2008).

Since Lake Kinneret is located in a semiarid climate zone and extensively used for drinking water supply, it experiences large annual water level fluctuations (Zohary and Ostrovsky 2011). The variability in water level has an important impact on the shore vegetation. The occurrence of plants species is determined by the sediment's properties (Gasith and Gafny 1990). Vegetation cover increases with an increase in the area of exposed shelf (Gafny and Gasith 2000). The littoral vegetation is an integral part of the ecosystem of the lake since it decreases erosion and serves as a habitat for other organisms (Zohary and Gasith 2014). When the vegetation is inundated, it decreases the wave’s energy and protects the habitats in the shallow water from strong currents (Zohary and Gasith 2014). The common composition of Lake's Kinneret littoral vegetation is characterized by *Phragmites australis* (Cav.) Trin. ex Steud. (common reed), *Typha angustata* Bory & Chaub. (cattail or bulrush), *Cyperus articulatus*, *C. alopecuroides* Rottb. 1773 (sedge), and the tree *Tamarix jordanis* Boiss. (tamarisk).

## Methods

### Sampling sites

**Fig. 1:**
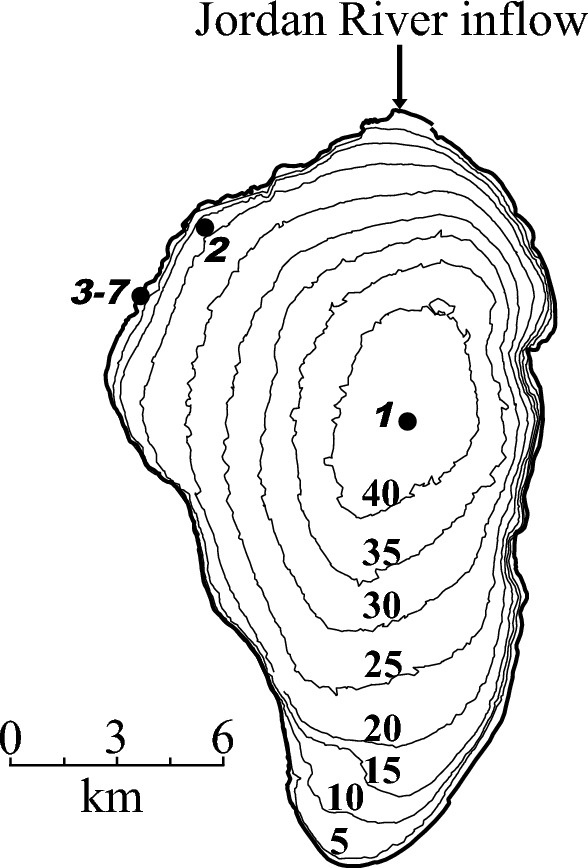
Map of Lake Kinneret with sampling sites. The values in the plain font stand for the water depth. The values in the bold italic font stand for the sampling site (see Table 1)

For our study we selected 7 sampling sites along a transect from the central lake station (A) towards Kibbutz Ginosar, located on the Northwestern shore of the lake. The exact sampling sites are detailed in Table 1 and Fig. 1. Sites 1 and 2 represent the open waters of the lake (Table 1). During the period of sampling, the sediment–water interface at Site 1 was exposed to anoxic sulfidic water while the overlaying water of Site 2 was always oxic. The water depths at Sites 1 and 2 were 37 m and 12 m, respectively (Table 1 and Fig. 1). The detailed report on the concentrations of oxygen, hydrogen sulfide, sulfide oxidation intermediates (SOIs), including zero-valent sulfur (ZVS), thiosulfate and sulfite, at 37 m water depths during the annual cycle of hydrographic conditions may be found in the work of Knossow et al. (2015). A detailed description of the chemical structure of the chemocline with an emphasis on the sulfur cycling as available from Avetisyan et al. (2019).

**Table 1:** Description of sampling sites including sampling dates, coordinates, water depths and information regarding the presence ( +) or absence ( −) of vegetation

Sampling Site	Date	Coordinates	Water depth, m	Distance from the shore, m	Vegetation
1	08/01/2015	32.8151°N 35.6061°E	37	n.a	–
2	01/12/2015	32.8543°N 35.5527°E	12	n.a	–
3	26/10/2015	32.8462°N 35.5270°E	0.1–0.2	n.a	–
4	15/05/2016	32.8453°N 35.5273°E	0.1–0.2	n.a	+
5	09/08/2015	32.8469°N 35.5258°E	0	ca. 5	–
6	16/06/2015	32.8467°N 35.5258°E	0	ca. 20	+
7	12/11/2014	32.8478°N 35.5254°E	0	ca. 40	+

n.a. : not applicable

Sites 3–7 were located in the littoral zone (Table 1, Fig. 1) at the beach kibbutz Ginosar, which is characterized by fluvial sediments. Sites 3 and 4 were overlaid by 10–20 cm of oxic waters, while Sites 5–7 were situated above the water level. During the high-water level periods (starting in February), the vegetation provides cover, sites for colonization, and food for the littoral biota until it decomposes (Gasith and Gafny 1990). The long term (in the order of decades) fluctuations in the lake water level results in the development of inundated vegetation. Moreover, the littoral sediment surface is affected by advection, which is a result of wave breaking, leading to transport of reduced solutes from the anoxic sediment to the oxic water column (Hofmann et al. 2010). The pore-waters of Lake Kinneret are also affected by the advecting groundwater brines along the western shore. The shore vegetation is poorly diverse, containing mostly bulrushes of the species *C. articulatus* and bushes. The presence of vegetation is usually seasonal with high inter-annual variability.

### Sampling

**Fig. 2:**
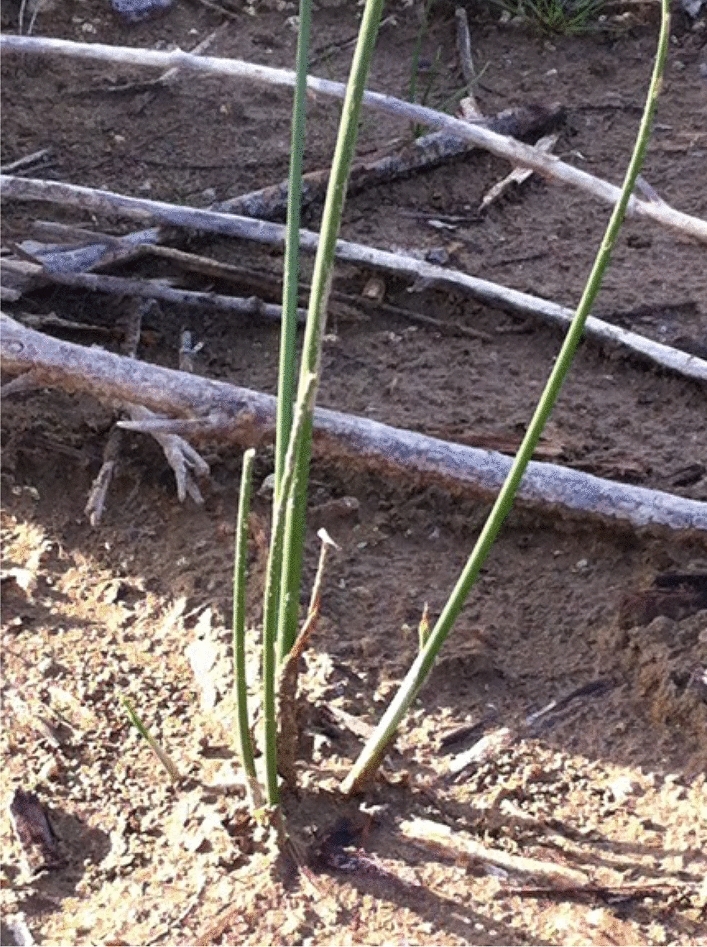
*Cyperus articulatus*, cordgrass, which was sampled in all vegetated cores, on the shore of Lake Kinneret

Sampling was conducted in this area on 5 different dates (Table 1). Sediments were collected at 0–20 cm water depth and on the dry land, ca. 20 m from the waterfront. Vegetated sediments contained *C. articulatus* (Fig. 2). Sampling on the shore and at < 0.2 m water depth was performed manually by hammering 60 cm long aluminum core liners with a diameter of 9.5 cm into the sediment and digging them out by shovel. The cores were immediately closed with stoppers, which were secured with duct tape. At the deeper sites, the cores, which were 40 cm long with a diameter of 5 cm, were retrieved by gravity corer from the RV Hermona (KLL).

During each sampling, triplicate cores were retrieved from the same location. These cores were used for: (1) pore-water analysis; (2) solid phase analysis; and (3) root content analysis. Preservations for solid phase analyses were performed immediately after sampling. Pore-water extraction and sample preservation were performed in less than 30 h after sampling. Roots content was measured in less than 48 h after sampling.

### Analytical methods: pore waters

Pore-water was extracted under nitrogen atmosphere in the glove-bag using tube samplers with 9 cm long, 4.5 mm diameter and mean pore size of 0.15 μm (MacroRhizon, Rhizosphere Research Products, the Netherlands) for the cores from the shore sites and tube samplers with 5.5 cm long, 1 mm diameter and mean pore size of 0.15 μm (MicroRhizon, Rhizosphere Research Products, the Netherlands) for the cores from the deepwater sites. Pore-water was extracted in 4 cm intervals. Rhizons were soaked in anoxic water for 40 min before sampling. A 60 mL syringe with a three-way Luer-lock connector was attached to Rhizon to extract 30–50 mL of pore-water from each depth. The syringe was washed with the first milliliter of each pore-water sample to avoid contamination of the sample. Pore-waters from each depth were transferred into falcon tubes inside the glove bag and immediately processed or preserved for further analyses.

Pore-water for sulfate quantification was preserved by mixing with 20% zinc acetate and stored refrigerated. Measurement of SO_4_^2−^ was performed by ion chromatography using DIONEX, DX500 chromatograph equipped with AG4A-SC guard column, and AS4A-SC anion exchange column. The method detection limit (MDL) is 10 µM (Dionex application note). An aliquot of the sample, which was used for sulfate quantification, was used for measurement of hydrogen sulfide at concentrations > 1 μM by spectrophotometry (Cline 1969) with MDL of 1 µM.

Measurement of cyanide-reactive ZVS was performed according to Kamyshny (2009). This method accounts for sum of dissolved, colloidal and polysulfide zero-valent sulfur. The samples (1–10 mL) were added to 20 mL of boiling 1% boric acid solution and 20 µL of 10% KCN solution were added. The solution was boiled for reaction completion and sample pre-concentration. Thiocyanate was quantified according to Rong et al. (2005) by HPLC (1260 Infinity, Agilent Technologies, Waldbronn, Germany) using a polyethylene glycol modified reversed-phase C30 column (Develosil 5 μm RPAQUEOUS, 150 × 4.6 mm I.D, Nomura Chemical, Seto, Japan) and an eluent composed of 300 mM sodium sulfate and 50 mM sodium chloride. The flow rate was 1.0 mL min^−1^ and UV detection was set at a 220 nm wavelength in order to quantify thiocyanate with the detection limit of 0.06 µM.

Quantification of thiosulfate, sulfite, and hydrogen sulfide at concentrations < 1 μM was performed by derivatization with monobromobimane at room temperature (Blonder et al. 2017; Fahey and Newton 1987; Zopfi et al. 2008; and references therein). The formed derivatives were quantified by HPLC (1260 Infinity, Agilent Technologies, Waldbronn, Germany) using a C18 reverse phase column (Prevail C18, 5 μm, 250 × 4.6 mm, Grace, Columbia, MD, USA). The eluent was composed of a varying gradient of 100% methanol (eluent A) and 0.25% (v/v) acetic acid solution adjusted to pH 3.5 with 5N NaOH (eluent B) at a flow rate 1 mL min^−1^. The gradient program was as follows: start 10% A, 14 min 12% A, 30–38 min 30% A, 54 min 42% A, 82 min 80% A, 84–88 min 100% A, 90–95 min 10% A. Fluorescence (excitation at 380 nm, emission at 480 nm) was used to quantify the bimane derivatives. The detection of this method is 0.01 µM.

Quantification of total manganese (Mn_TOT_) was performed by spectrophotometry according to Goto et al. (1977). The MDL of this method is 0.7 μM. Total dissolved iron and dissolved Fe(II) concentrations were quantified by spectrophotometry using the method of Stookey (1970) with and without reduction of Fe(III) with ascorbic acid, respectively. The MDL of this method is 0.5 μM. Pore-water pH was measured by a calibrated pH-meter (250Aplus, Thermo Orion, Beverly, MA, USA) with pH electrode (E 6384, Sigma, St. Louis, MO, USA).

## Analytical methods: solid phase

Core extruders were used to extract the sediment from the core liners. The sediment was cut into 2 cm thick slices. The slices were divided into subsamples for preservation for various analyses. For porosity analysis, ca. 10 mL of sediment was placed in falcon tube and stored at 4 °C. For ZVS, acid volatile sulfide (AVS, mostly FeS) and chromium reducible sulfur (CRS, mostly pyrite, FeS_2_) analysis, 25 mL of sample was added to a falcon tube with 25 mL of 5% zinc acetate solution. The samples were stored in the freezer. For iron and manganese analyses, 40 mL of sediment sample were preserved in the freezer without pre-treatment.

Pure methanol was used for extraction of ZVS from the sediment, which was pretreated with zinc acetate. 400 mL of methanol was added to 12–16 g of pretreated sediment in 1 L glass bottle. The samples were shaken for 16 h on a rotary shaker. Quantification of ZVS was performed by HPLC–UV (Zopfi et al. 2004), with detection at 230 nm wavelength. The MDL is 10 μmol kg^−1^ (wet sediment).

Quantification of AVS and CRS was performed by a distillation (Fossing and Jørgensen 1989). Prior to the distillation, the sediment was extracted with methanol to avoid partial reduction of ZVS. The first step included boiling of the sediment with 20 mL 5 M HCl for two hours. H_2_S was collected in 15 mL of 5% zinc acetate traps. For the second step, the traps were changed, and the sample was boiled for three hours with 20 mL of 1 M acidic CrCl_2_ solution. Quantification of the H_2_S in the traps was performed according to Cline (1969). The MDL was 10 μmol kg^−1^ (wet sediment). Sulfur-bound iron (Fe_SB_) was defined in this work as sum of AVS and half of CRS.

Measurements of total iron (Fe_TOT_) and manganese were performed by ashing of 1–2 g wet sediment for 8 h at 450 °C in the muffle oven. A sample of 0.6 g of ashed and grounded sediment was placed in a 15 mL falcon tube and heated to near boiling temperature with 10 mL of 6 N HCl in a water bath for 24 h (Aller et al. 1986). The sample was centrifuged and the concentrations of iron and manganese in the sample were measured according to Stookey (1970) for Fe_TOT_ and Goto et al. (1977) for Mn_TOT_.

The term “highly reactive iron” (Fe_HR_) is defined as the fraction sedimentary iron which is reactive towards hydrogen sulfide or has already reacted with it (e.g., Fe_SB_) (Canfield 1989). The sequential extraction procedure was used to quantify Fe_HR_ (Poulton and Canfield 2005): (1) Fe_Mg_ was extracted with magnesium chloride solution, accounts for the adsorbed Fe(II), which was always below the detection limit; (2) Fe_ac_ was extracted with sodium acetate solution, accounts for Fe(II) carbonates, e.g., siderite and ankerite; (3) Fe_hydr_ was extracted with hydroxylamine hydrochloride solution, accounts for lepidocrocite and ferrihydrite; (4) Fe_dith_ was extracted with sodium dithionite solution, accounts for goethite, hematite, akaganéite; (5) Fe_ox_ was extracted with ammonium oxalate/oxalic acid solution, accounts for magnetite. Quantification of extracted iron in the liquid phase was performed by spectrophotometry (Stookey 1970). Fe_HR_ was defined as the sum of the results of all sequential extractions and of Fe_SB_.

Total organic carbon (TOC) was analyzed by Arnie Miller Laboratories Ltd. (Beer Sheva) by the Walkley–Black titration and colorimetric method according to the protocol from the Food and Agriculture Organization of the United Nations. The analysis was performed on the dry sediment after the removal of the shells, gravel, and plant roots.

### Biological and physical parameters

Analysis of roots content was performed on slices of sediment at 2 cm intervals. The sediment was sliced with a knife. The slices were weighed and washed to collect the roots, and the roots were weighed. The fraction weight of roots in the sediment slice (in weight %) indicates the roots content.

Porosity was analyzed by weighing 3–5 g of sediment sample in a pre-weighed cylinder. Milli-Q were added to the cylinder with the sediment until it was filled up to 10 mL and weighed. The sediment was dried in an oven for one week at 60 °C and weighed again. The calculation for porosity was based on the difference between volumes and weights of wet and dry sediment.

## Results

### Sites 1 and 2: sediment and pore-water composition in the profundal zone

**Fig. 3:**
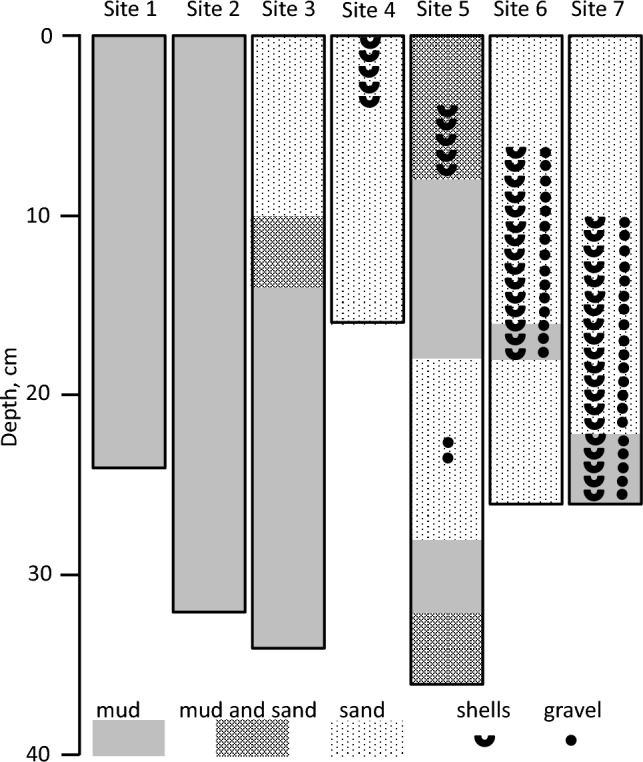
Vertical profiles of sediment substrata

**Fig. 4:**
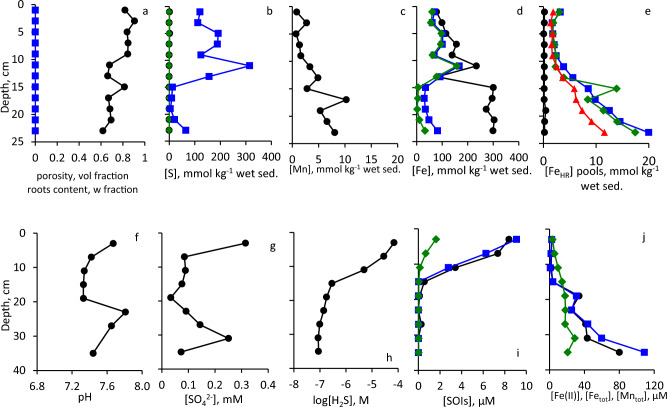
Vertical profiles of the solid phase and of the pore-water of sediments sampled from Site 1: **a** Porosity (black circles) and roots content (blue squares), **b** AVS (black circles, CRS (blue squares) and ZVS (green diamonds), **c** Mn_TOT_, **d** Fe_TOT_ (black circles), Fe_HR_ (blue squares), Fe_SB_ (green diamonds), **e** Fe_ac_ (black circles), Fe_hydr_ (blue squares) Fe_dith_ (green diamonds); Fe_ox_ (red triangles), **f** pore-water pH, **g** pore-water sulfate, **h** pore-water hydrogen sulfide (note the log-transformed X-scale), **i** pore-water SOIs: ZVS (black circles), thiosulfate (blue squares) and sulfite (green diamonds), **j** dissolved Fe(II) (black circles), Fe_TOT_ (blue squares) and Mn_TOT_ (green diamonds)

**Fig. 5:**
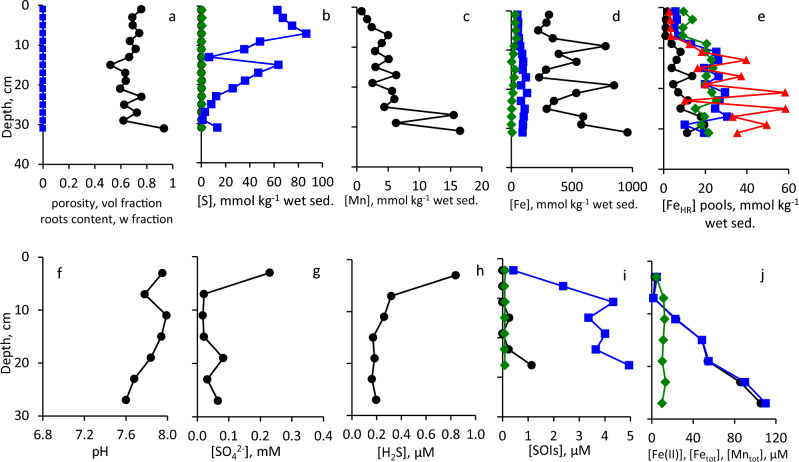
Vertical profiles of the solid phase and of the pore-water of sediments sampled from Site 2. Symbols and colors in panel (**a**–**j**) are the same as in Fig. 4

Sediments at these sites consist of homogenized cohesive silt–clay (Fig. 3). Eckert (2000) reported that composition of the sediments at Site 2 is more heterogeneous than at Site 1 due to proximity to the shore. Physical and chemical parameters at Sites 1 and 2 (37 m water depth, sulfidic deep waters, and 12 m water depth, oxic water column, respectively) are presented in Fig. 4, 5. Porosity is relatively high, and no plant roots were detected (Fig. 4a, 5a). TOC contents in surface sediments of both sites were < 1% of dry sediment weight (Table S1). Contents of AVS and ZVS are very low, while CRS content is high and generally decreases with sediment depth (Fig. 4b, 5b). Mn content increases with depth at both sites to > 10 mmol kg^−1^ (Fig. 4c, 5c). At Site 1, in the upper 12 cm of the core, most of the Fe_TOT_ and Fe_HR_ are present as Fe_SB_, while below this depth, Fe_HR_ content is low and only traces of Fe_SB_ are present (Fig. 4d). At Site 2, most of the iron does not belong to the highly reactive pool throughout the core (Fig. 5d). Fe(III) (hydr)oxides prevail over Fe(II) carbonates in the sediments with Fe_hydr_ and Fe_dith_ being the most abundant pools at Site 1 (Fig. 4e) and Fe_ox_ at Site 2 (Fig. 5e). Fe_Mg_ was below the detection limit in all samples from both sites. Pore-waters are slightly basic with pH in 7.3–8.0 range (Fig. 4f, 5f). Sulfate concentrations are similar to or lower than those found in the hypolimnion of the lake (Knossow et al. 2015) and range between 18 and 315 μM (Fig. 4g, 5g). Concentration of hydrogen sulfide decreases from 71 μM in the surface sediments to 0.085–0.29 μM at 13–35 cm depth at Site 1 (Fig. 4h). At Site 2, which is overlaid by oxic waters, concentrations of hydrogen sulfide do not exceed 0.84 μM even in the surface sediments (Fig. 5h). At Site 1, the concentrations of sulfide oxidation intermediates (SOIs, zero-valent sulfur, thiosulfate and sulfite) decrease with depth as well. Thiosulfate and ZVS are more abundant than sulfite; their concentrations in the surface sediments reach 9.1 μM, 6.2 μM, and 1.6 μM, respectively (Fig. 4i). At Site 2, thiosulfate prevails over other SOIs, and its concentration increases with depth (Fig. 5i). The concentration of dissolved manganese increases at both sites with depth and reaches 29 μM at the Site 1 (Fig. 4j, 5j). Sharp increase in both total and dissolved iron in the pore-waters starts below 15 cm depth at Site 1 and below 7 cm at Site 2. Concentration of iron in pore-waters at both sites is > 100 μM at the bottom of the core (Fig. 4j, 5j).

### Sites 3 and 4: Sediment and pore-water composition in the littoral sediments

**Fig. 6:**
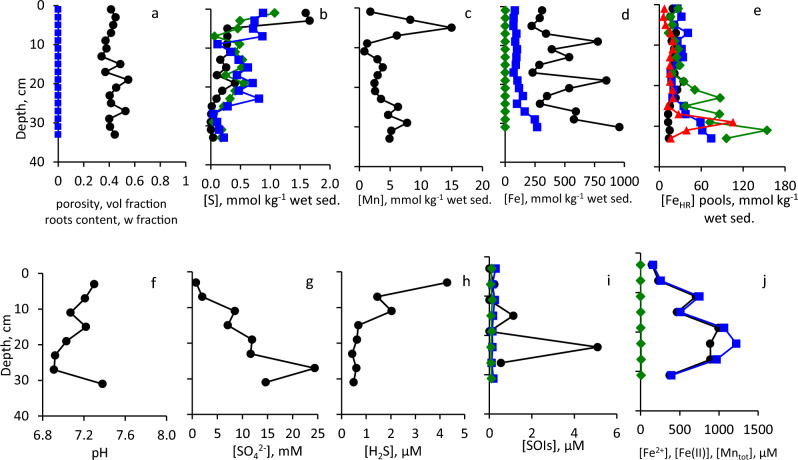
Vertical profiles of the solid phase and of the pore-water of sediments sampled from Site 3. Symbols and colors in panel (**a**–**j**) are the same as in Fig. 4

**Fig. 7:**
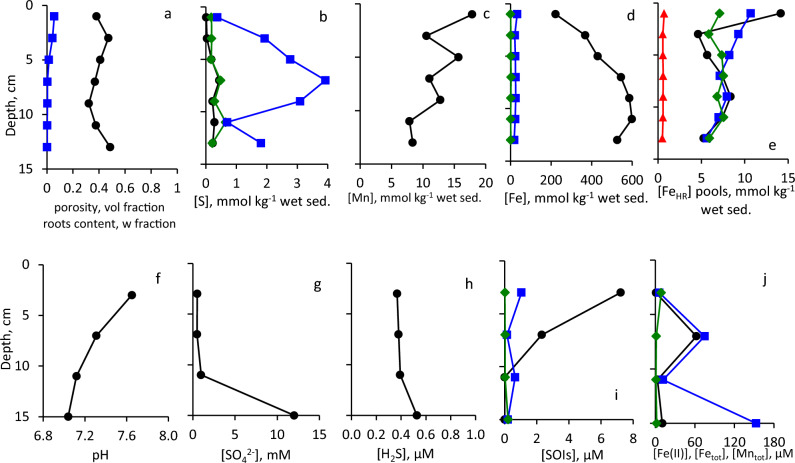
Vertical profiles of the solid phase and of the pore-water of sediments sampled from Site 4. Symbols and colors in panel (**a–j**) are the same as in Fig. 4

At Site 3, in the upper 6 cm sediment consists of sand with shells, at 6–10 cm of sand without shells, at 10–14 cm of sand-mud mixture and of cohesive mud below this depth (Fig. 3). At Site 4, the whole core, 14 cm in length, consists of sand with shells in the upper 4 cm of the core (Fig. 3). Physical and chemical parameters at Sites 3 and 4 (10 cm water depth, barren and vegetated sediments, respectively) are presented in Fig. 6 and 7. Porosity is relatively low, and no plant roots were detected in the barren sediments (Fig. 6a, 7a). TOC contents were higher at Site 3 than at Site 4 (Table S1). In the vegetated sediments, roots content was 5.6 weight % in the upper two centimeters of the sediment and decreased to < 1% at 6–8 cm sediment depth, while below 10 cm roots this content was < 0.3% (Fig. 7a). The contents of reduced sulfur species in the sediments are extremely low, with CRS content lower by two orders of magnitude as compared with in the sediments overlaid by deeper waters (Fig. 6b, 7b). Mn content is similar to those in the open water sediments, but its highest content was detected at or just below the sediment water interface (Fig. 6c, 7c). Fe_TOT_ contents are similar to those at the open water sites (Fig. 6d, 7d). In the barren sediments, the highly reactive iron content is higher than in the vegetated sediments, while sulfur-bound iron concentrations do not exceed 2.5 mmol kg^−1^ wet sediment (Fig. 6d, 7d). In the vegetated sediments and in the upper 20 cm of the barren sediments, the concentrations of all highly reactive iron pools are similar, while below this layer the least reactive phases prevail (Fig. 6e, 7e). Fe_Mg_ was below the detection limit in all samples from both sites. Pore-waters pH in sediments at these sites are lower than in the open water sediments, and in vegetated sediments pH decreases with depth (Fig. 6f, 7f). Sulfate concentrations sharply increase below 10 cm sediment depth to concentrations, which are ca. 50 times higher than in the epilimnion of the lake, most likely due to an advection of saline waters through the sediments (Mortimer et al. 1999; Rimmer and Gal 2003; Fig. 6g, 7g). Hydrogen sulfide concentrations are in the low micromolar range in barren and < 1 μM in the vegetated sediments (Fig. 6h, 7h). At these sites, the concentrations of sulfur oxyanions are very low as well, while the concentration of ZVS in pore-waters is higher than concentration of hydrogen sulfide (Fig. 6i, 7i). Concentrations of iron in the pore-waters is much higher than at the open water sites, especially in the barren sediments, where total pore-water iron reaches 1.22 mM, while concentrations of dissolved manganese are much lower than in the open water sediments (Fig. 6j, 7j).

### Sites 5–7: sediment and pore-water composition of the onshore sediments

**Fig. 8:**
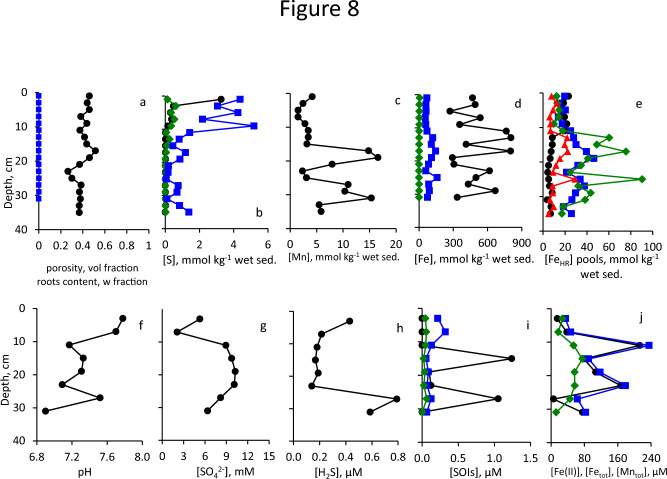
Vertical profiles of the solid phase and of the pore-water of sediments sampled from Site 5. Symbols and colors in panel (**a**–**j**) are the same as in Fig. 4

**Fig. 9:**
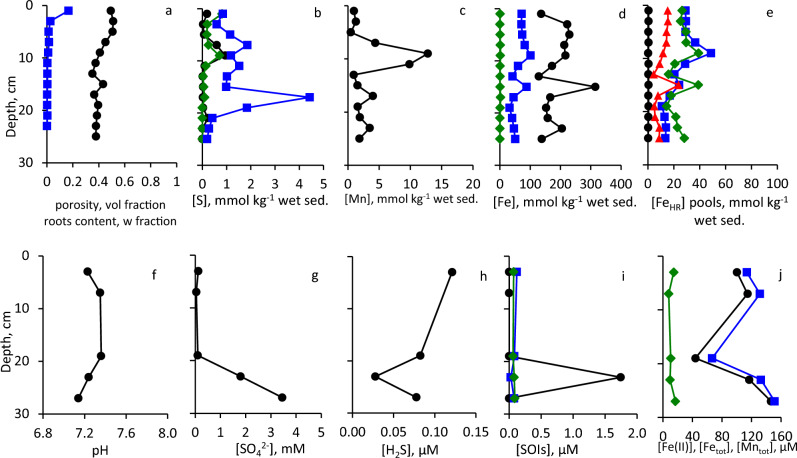
Vertical profiles of the solid phase and of the pore-water of sediments sampled from Site 6. Symbols and colors in panel (**a**–**j**) are the same as in Fig. 4

**Fig. 10:**
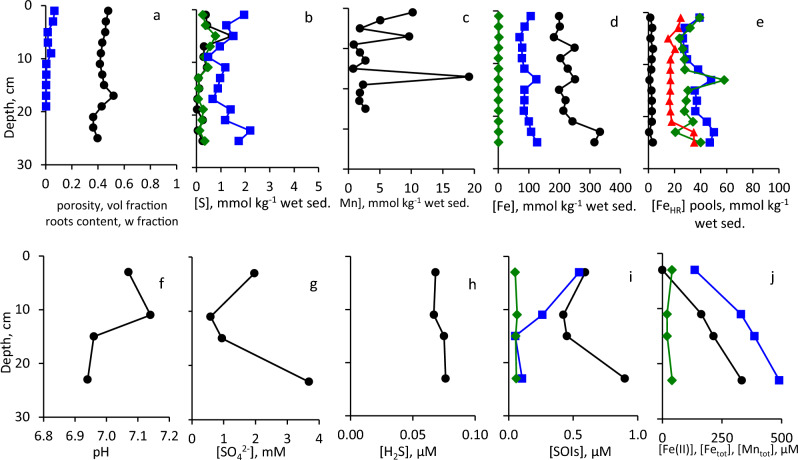
Vertical profiles of the solid phase and of the pore-water of sediments sampled from Site 7. Symbols and colors in panel (**a–j**) are the same as in Fig. 4

At Site 5, upper 8 cm of the sediment consists of sand-mud mixture with reddish-brown inclusions, at 8–18 cm sediment consists of the cohesive mud with shells at 8–12 cm depth. At 18–28 cm, the sediment was sandy with a layer of gravel at 22–24 cm, while at 28–32 cm the sediments consisted of cohesive mud, and of sand-mud mixture below this depth (Fig. 3). At Site 6, the sediments are mostly sandy with gravel and shells at 6–18 cm depth and muddy layer at 16–18 cm (Fig. 3). At Site 7, sediments are sandy down to 22 cm depth with muddy layer below this depth, while shells and gravel were present in the core below 10 cm depth (Fig. 3). Physical and chemical parameters at Sites 5, 6 and 7 (on shore, barren sediment, and vegetated sediment at high and low tide, respectively) are presented in Fig. 8, 9 and 10. Porosity at these sites is low and similar to the neighboring sites covered with water (Fig. 8a, 9a, 10a). TOC contents in sediments of all sites were < 1.3% of dry sediment weight except for one horizon at Site 6 (Table S1). At both vegetated sites the root content is > 1% by weight above 10 cm sediment depth and lower below this depth (Fig. 8a, 9a, 10a). The content of reduced sulfur species in the sediments is extremely low and CRS is the dominant reduced sulfur pool (Fig. 8b, 9b, 10b). Mn contents are similar to those in the open water sediments (Fig. 8c, 9c, 10c). Fe_TOT_ contents are higher in the barren sediment, while highly reactive iron contents are similar in all cores and sulfur-bound iron contents are low, similarly to sediments covered by shallow waters (Fig. 8d, 9d, 10d). At all sites, the least reactive fractions of highly reactive iron prevail in the sediments (Fig. 8e, 9e, 10e). Fe_Mg_ was below the detection limit in all samples from these sites. The pore-waters pH in sediments at these sites is in the range of 6.9–7.8 (Fig. 8f, 9f, 10f). Sulfate concentration sharply increases below 10–20 cm sediment depth, similarly to the sites covered by shallow water (Fig. 8g, 9g, 10g). Hydrogen sulfide concentrations in the barren sediments are < 0.8 μM and in the vegetated sediments are < 0.15 μM (Fig. 8h, 9h, 10h). The concentrations of sulfur oxyanions are very low as well, while the concentration of ZVS in some samples exceeded 1 μM (Fig. 8i, 9i, 10i). The concentrations of iron in the pore-waters are higher than at the open water sites, but lower than in the sediments covered by shallow waters, while concentrations of dissolved manganese are similar to those in the open water sediments (Fig. 8j, 9j, 10j).

## Discussion

### Comparison between open water sites covered with sulfidic and oxic waters

Characteristics of open water sites, Site 1 (37 m water depth) and Site 2 (12 m water depth) are compared in Table 1 and Fig. 4, 5, and 11a,b. At both sites sediments consist of cohesive mud (Fig. 3). Increase in Fe_TOT_ and Fe_HR_ as well as decrease in Fe_SB_/Fe_HR_ below 15 cm depth are attributed by Eckert (2000) to the drainage of Hula valley, a former wetland, drained in the 1950’s. Sediments below 15 cm at Site 2 contain more reactive Fe_TOT_ and Fe(III) (hydr)oxides than Site 1 as it is located close to mouth of the Jordan river and thus was more affected by a sedimentary input from the Hula valley (Fig. 4d,e, 5d,e). In the upper sediment zone, sulfate reduction leads to a depletion of sulfate at 10–22 cm sediment depth (Fig. 4g, 5g). Hydrogen sulfide at Sites 1 and 2 becomes depleted in the sediments at 15 cm and 7 cm sediment (Fig. 4h, 5h), respectively, and ferruginous-manganous pore-waters are present below this horizon (Fig. 4j, 5j) due to a depletion of sulfate available for microbial sulfate reduction. Dissolved manganese appears in the pore-waters at lower depths than dissolved iron due to its lower affinity to hydrogen sulfide and relatively high solubility of MnS.

**Fig. 11:**
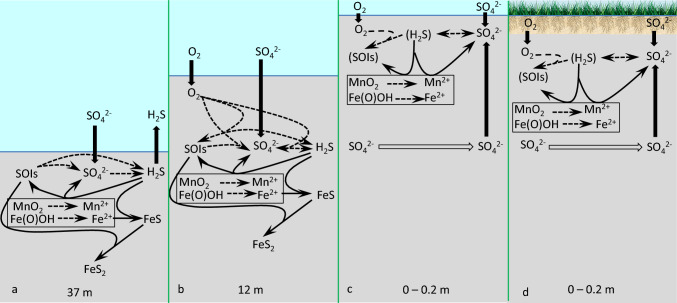
Scheme of inorganic sulfur and iron cycling in the sediments of Lake Kinneret at various hydrographic settings. Thick full arrows represent diffusion. Thick empty arrows represent lateral advection of sulfate. Thin solid arrows represent chemical processes, while thin dashed arrows represent processes, which may be microbially enhanced. Parentheses indicate compounds which present at low concentrations. Processes which lead to formation of these compounds cannot be fully clarified based on the results of this work (see text for details). Panel (**d**): desiccation results in similar or even strong oxygenation of the surface sediments than roots penetration

Nearly all reactive sulfur is present in the form of CRS. The process of pyrite formation from AVS and ZVS is fast enough to result in presence of only trace amounts AVS and ZVS in the sediments (Fig. 4b, 5b). Oxidation of hydrogen sulfide with Fe(III) (hydr)oxides results in the formation of relatively high concentrations of SOIs, especially of thiosulfate. At Site 2, concentrations of thiosulfate in pore-waters are even higher than concentrations of hydrogen sulfide that is not unusual for low-sulfide pore-waters in the presence of high amount of Fe_HR_ (Blonder et al. 2017).

Detailed study of sulfur and iron speciation in the sediments of the same open water sites was performed earlier by Eckert (2000) with somewhat different methodology, while manganese speciation and concentrations of SOIs in pore-waters were not reported in Eckert (2000), Sites 1 and 2 are referred as stations A and S, respectively). Despite these differences, direct comparison between parameters measured in both works is possible. Our results agree well with the results presented by Eckert (2000) except for the concentrations of AVS and ZVS, which were in both cores lower by one to two orders of magnitude than those reported by Eckert (2000).

### Influence of location: comparison between open water, littoral zone and onshore barren sites

Here we compare speciation of the redox-sensitive elements at Sites 1 (37 m water depth), 3 (barren site covered by < 20 cm of water) and 5 (barren site not covered with water) (Table 1, Fig. 4, 6, 8 and 11a,c,d). Site 5 was sampled in August, during the hottest period of the year, when the sediments are desiccated without inundation. At Sites 3 and 5, the redox conditions are very different from the conditions at Site 1, where hydrogen sulfide is present at high concentrations in the surface sediments while dissolved iron is absent. At Sites 3 and 5 only a minor fraction of Fe_TOT_ is present as Fe_HR_, while Fe_SB_ is either extremely low or totally absent in these sediments (Fig. 4d, 6d, 8d). On the other hand, speciation of highly reactive iron is similar at all sites, with prevailing of the most reactive poorly crystalline Fe(III) (hydr)oxide forms (Fig. 4e, 6e, 8e). Presence of relative high contents of freshly precipitated and thus readily bioavailable iron results in formation of ferruginous pore-waters already at 3 cm sediment depth even at Site 5, despite absence of water above it and desiccation of the top sediment layer due to hot weather in August. Comparison between Sites 3 and 5 shows that the presence of sandy and muddy layers affects speciation of the redox-sensitive elements more than the presence or absence of shallow water layer above the sediment. In the muddy layers concentrations of dissolved iron are usually higher than in the sandy layers, while the trend of dissolved hydrogen sulfide is opposite (Fig. 6h,j and 8h,j). Relatively high concentrations of manganese and highly reactive iron at Sites 3 and 5 are associated with muddy sediments. Another increase in Mn_TOT_ is present near the sediment surface at Sites 3 and 5 due to oxidation of dissolved manganese, which diffuses from the deeper sediment layers.

Despite the presence of cohesive sediment, high TOC contents at Site 3 and high sulfate concentrations, hydrogen sulfide does not accumulate in the pore-waters of sediments at both Sites 3 and 5. The reason is most likely the high concentrations of Fe(III) (hydr)oxides, which can either suppress microbial sulfate reduction or oxidize hydrogen sulfide, resulting in so-called “cryptic” sulfur cycling. Such settings were documented in various marine sediments, including salt marshes, Arctic fjords, Gulf of Aqaba and deltaic sediments (Aller et al. 2010; Blonder et al. 2017; Boyko et al. 2019, 2021; Mills et al. 2016; Wehrmann et al. 2017). On the other hand, this type of cryptic sulfur cycle is much less studied in limnic environments. Although no direct measurements of microbial sulfate reduction were performed in this study, indirect evidence, such as oxygen isotope composition of sulfate, the presence of sub-micromolar concentrations of hydrogen sulfide and its oxidation intermediates as well as the presence of pyrite may be invoked to show its presence (Blonder et al. 2017).

The presence of cryptic sulfur cycle in the littoral sediments of Lake Kinneret is supported by the following lines of evidence: presence of traces of hydrogen sulfide (Fig. 6h, 8h), presence of its oxidation intermediates (Fig. 6i, 8i) and of CRS (Fig. 6b, 8b). Although there are certain similarities in speciation of iron and sulfur in the littoral sediments and sediments at Site 1 at > 15 cm sediment depth, detailed assessment of data shows that biogeochemical cycling of these elements is very different. Although below 15 cm depth at Site 1, the concentrations of H_2_S are in the 0.09–0.18 μM range (Fig. 3h) and the total sulfur in SOIs is ≤ 0.15 μM. On the other hand, at Sites 3 and 5 higher concentrations of ZVS, up to 5.0 and 1.2 μM, respectively, are present in the sediments. Another difference is much higher concentrations of highly reactive iron in the solid phase (Fig. 4d, 6d, 8d) and dissolved iron in the pore-waters at Sites 3 and 5 (Fig. 4j, 6j, 8j). We interpret these differences as an absence of sulfur cycling below 15 cm depth of the sediments at Site 1 as opposite to the presence of active, although cryptic, sulfur cycling in the littoral zone sediments.

### Comparison between littoral barren and vegetated sites

Here we compare speciation of the redox-sensitive elements at littoral water-covered sediments of Sites 3 and 4 (barren and vegetated, respectively, Table 1 and Fig. 6, 7 and 11c,d). The distance between the two sites was 104 m.

As at Site 4 only 16–18 cm long cores were retrieved, comparison with Site 3 is provided for this depth interval. It is impossible to evaluate impact of permeability on the cycling of the redox-sensitive elements at Site 4, as the whole core from the Site 4 is sandy. Site 4 differs from all other sites by very low Fe_HR_ content. Interestingly, this decrease is due to lower content of Fe(III) (hyrd)oxides, while the content of Fe(II) carbonates (siderite and ankerite) is similar to that at the other sites (Fig. 7e). Lower content of Fe(III) (hydr)oxides leads to relatively low concentrations of dissolved iron in the pore-waters at this site (Fig. 7j).

Although roots penetration is known to decrease pore-water pH, we have not detected such an effect in the shore sediments of Lake Kinneret. In the sediments, in contrast, pH is lower at the barren site (Site 3). Oxygen penetration into the upper layers of sediment can be deduced from various trends. First, while in the surface sediments of Site 3, hydrogen sulfide concentration in the pore-water was as high as 4.3 μM, at Site 4 its concentration is lower by an order of magnitude (Fig. 6h, 7h). One of the parameters, which may decrease oxygen penetration depth and result in formation of reducing conditions is higher TOC content in the sediments at Site 3 than at Site 4. Secondly, the concentrations of the SOIs are higher in the upper sediments of core at Site 4 (Fig. 6i, 7i), most likely due to the higher rates of hydrogen sulfide oxidation associated with the penetration of roots into the surface sediments. The concentrations of both dissolved Fe(II) and total dissolved iron in the surface sediments are as well lower by 1–1.5 orders of magnitude at Site 4 than at Site 3 (Fig. 6j, 7j). Dissolved iron may be formed either by microbial reduction of reactive Fe(III) minerals or by their chemical reduction with hydrogen sulfide (Lovley 2004; Thamdrup 2000; Thamdrup et al. 1994;). As at Site 4 concentrations of both reactive Fe(III) species and hydrogen sulfide are relatively low, these processes should be kinetically hindered. While sharp increase in total Mn is observed at 4–6 cm depth in the sediment at Site 3, most likely due to a presence of the redoxcline at this depth, no such feature was characteristic for Site 4, and in the upper 10 cm of the sediment, the content of total Mn at Site 4 is similar to the highest content at Site 3.

Fast oxidation of hydrogen sulfide at Site 4 is clear from the high concentrations of SOIs, with concentration of dissolved ZVS and thiosulfate 20 and 3 times higher than that of H_2_S, respectively. Roots penetration in the upper 10 cm of the sandy sediments may result in formation of highly non-homogeneous redox conditions in the sediments: oxic niches in the vicinity of the roots may be neighbored by locally anoxic sediments, in which sulfate, iron and manganese reduction takes place (Koop-Jacobsen and Wenzhofer 2015; Zhu and Aller 2013). Such conditions result from fast cycling of redox-sensitive elements and, due to fast reoxidation of hydrogen sulfide, leading to cryptic sulfur cycling.

### Comparison between onshore barren and vegetated sites

Here we compare speciation of the redox-sensitive elements in the sediments of Sites 5 and 6 which are not covered by water (barren and vegetated, respectively, Table 1 and Fig. 8 and 9). The distance between sites was 222 m. Sites 5 and 6 were compared for upper 26–28 cm (length of the cores at Site 6). In both cores there is a muddy layer in the middle, which is thicker at Site 5 (Fig. 3). The presence of sand-mud transitions in both cores allows evaluation of the influence of the sediment permeability on the speciation of the redox-sensitive elements in the cores.

At Site 6, similarly to Sites 3 and 5, hydrogen sulfide concentrations in pore-waters locally increase in the sandy sediments (Fig. 6h, 8h, 9h), while dissolved iron concentrations are lower in the sandy sediments and at sand-mud boundaries (Fig. 6j, 8j, 9j). In the surface sediments, the presence of the sandy layer leads to higher dissolved iron concentrations even in the presence of the roots, while other parameters, including concentrations of SOIs are not significantly affected by the presence of roots. Other possible explanation of the higher concentrations of dissolved iron in the surface sediments at Site 6 is that it was sampled in June, while Site 5 was sampled in August, when, due to the higher temperatures, desiccation cracks were visible on the sediment surface. Thus, we suggest that oxygen penetration to the sediments through the desiccation cracks is more important than bioturbation due to penetration of *C. articulatus*.

Comparison of these two pairs of sites show that roots penetration is not the most important factor controlling speciation of the redox-sensitive elements in the sediments. The main factor which controls their speciation is sediments permeability. While hydrogen sulfide is always higher in the muddy sediment, concentrations of dissolved iron depend on availability of reactive Fe(III) phases and most likely significantly varies during the annual cycle of hydrographic conditions and seasonal temperature variations.

### Influence of location: comparison between littoral zone and onshore vegetated sites

A detailed discussion on the environmental factors affecting cycling of the redox-sensitive elements in the sediments at Sites 4 and 6 was provided above (Table 1 and Fig. 7 and 9). Thus, in this part we compare only the upper 16 cm of the sandy sediment layer at these sites in order to pinpoint the influence of the presence of shallow water cover on the sediments, which are affected by the penetration of the roots. The distance between Sites 4 and 6 is 210 m and the sediments were sampled during the same season, May–June, when on-shore sediments are not significantly affected by the desiccation. The main feature, which is observed through comparison between the two sites, is a presence of the local maxima in the Mn_TOT_, Fe_TOT_ and Fe_HR_, especially its Fe_hydr_ fraction which accounts for the poorly crystalline iron (hydr)oxides, at 8–10 cm depth of the sediments at Site 6 (but not at Site 4). This feature is diagnostic for the redox boundary in the sediments and results from precipitation of metal oxides due to oxidation of dissolved Fe(II) and Mn(II) at the oxic-anoxic interface. On the other hand, the presence of high concentrations of dissolved iron above this horizon is incompatible with the presence of oxygen. Most likely, this feature appeared from the earlier season of the year and is not caused by the roots, which do not penetrate to the 10 cm sediment depth at this site. Comparison between these two sites leads to the conclusion that in the sediments overlaid with shallow water the impact of roots is not the main control on the redox conditions of the sediments, especially below the 5 cm sediment depth.

### Comparison between vegetated sites, which are situated above water level, during the high- and low-water level periods

In this section, the comparison of vegetated sediments, which are above water level during the high-water level period at Site 6 (Fig. 9) and low-water level period at Site 7 (Fig. 10) are discussed. The sites are situated 128 m apart from each other. Site 6 was sampled in June, while Site 7 was sampled in November (Table 1). The sediments at both sites are sandy with muddy layer, which is deeper and thicker at Site 7 (Fig. 3). Sites are situated very close to each other, thus during the low water level sampling, Site 7 was further away from the water column. Roots penetration is deeper at Site 7, possibly due to a seasonal factor. While speciation of the redox-sensitive elements at these sites is similar, dissolved iron concentrations in the pore-waters increase with depth and rich higher values below 10 cm depth in November than in June (Fig. 9j, 10j). On the other hand, near the sediment surface there is no dissolved Fe^2+^ in the pore-waters, likely due to high (5.8 w%) content of roots (Fig. 10a). The highest Mn content in the sediments is detected at 10 cm depth for Site 6 and at 15 cm depth for Site 7 (Fig. 9c, 10c). We suggest that at Site 7, similarly to Site 6, this feature appeared from the earlier season of the year.

Comparison between these sites provides further support to the assumption that annual cycle of climatic and hydrographic conditions, which affect, among other factors, roots penetration depth, provides to some extent a control on the sediment redox status. On the other hand, the seasonal desiccation of the sediments affects speciation of the redox-sensitive elements in barren sediments much stronger than in the vegetated sediments.

## Conclusions

At the deepest part of the lake, the concentrations of both hydrogen sulfide and sulfate decrease with sediment depth and below 15 cm transition between sulfidic and ferruginous pore-waters occurs, while dissolved manganese concentrations are higher than dissolved iron concentrations in the upper 15 cm of the sediment. In these sediments, which are overlaid by sulfidic waters, only Fe(III) (hydr)oxides are responsible for oxidation of hydrogen sulfide, while in the sediments overlaid by oxic waters, oxidation of hydrogen sulfide with oxygen takes place as well in the surface sediments (Fig. 11a,b).

Vegetated sediments are affected by penetration of *C. articulatus* roots, which is confined to the upper 10 cm of sediments. In the littoral sites affected by *C. articulatus* vegetation, sediment permeability has a more significant effect on speciation of iron, manganese, and sulfur than penetration of the roots of *C. articulatus*. At the sites, which are situated on the shore and are not covered with water, desiccation has similar or even stronger effect on speciation of the redox sensitive elements than bioturbation by roots penetration, especially during the hottest time of the year (e.g., August) due to enhanced oxygen transport into the upper sediment layer (Fig. 11c,d). The absolute water level in the lake has no direct effect on speciation of the redox-sensitive elements.

Although sulfate concentrations in the pore-waters of littoral sediments are much higher than at the deeper sites, concentrations of hydrogen sulfide are extremely low and never exceed 1 μM. On the other hand, presence of sulfur cycling in the shore sediments is supported by the indirect evidence: the presence of high concentrations of sulfate, relatively high concentrations of SOIs, and traces of solid reduced sulfur phases (ZVS, AVS, CRS). In the sediments at the littoral sites, pore-water chemistry is dominated by dissolved Mn and Fe in the surface sediments, while in the deeper sediment horizons dissolved Fe prevails over dissolved Mn as a result of high contents of highly reactive iron in the sediments of the lake. Thus, sulfur cycle in these sediments may be defined as “cryptic” (Fig. 11c,d). Further research is required to reveal the quantitative constraints of cryptic sulfur cycling in the littoral sediments of the lake.

## Supplementary Information

Below is the link to the electronic supplementary material.

Supplementary file1 (XLSX 44 kb)

## Data Availability

Raw data file which contains all raw data is available in the Supplementary Information.
